# Promoter Hypomethylation Unleashes HMGA1 to Orchestrate Immune Evasion and Therapy Resistance Across Cancers

**DOI:** 10.3390/biology14121758

**Published:** 2025-12-09

**Authors:** Iram Shahzadi, Taswar Ahsan, Shoaib Anwaar, Wajid Zaman, Houjun Xia

**Affiliations:** 1 Center for Cancer Immunology, Institute of Biomedicine and Biotechnology, Shenzhen Institutes of Advanced Technology, Chinese Academy of Sciences, Shenzhen 518055, China; iram@siat.ac.cn; 2 University of Chinese Academy of Sciences, Beijing 100049, China; 3 Institute of Plant Protection, Liaoning Academy of Agricultural Sciences, Shenyang 110161, China; taswar.micro@gmail.com; 4 Frazer Institute, Faculty of Health, Medicine and Behavioural Sciences, The University of Queensland, Brisbane, QLD 4102, Australia; s.anwaar87@gmail.com; 5 Department of Life Sciences, Yeungnam University, Gyeongsan 38541, Republic of Korea; 6 Faculty of Pharmaceutical Sciences, Shenzhen University of Advanced Technology, Shenzhen 518107, China

**Keywords:** HMGA1, pan-cancer, immunosuppressive tumor microenvironment, AKT inhibitor resistance, promoter hypomethylation, biomarker, Capivasertib

## Abstract

High mobility group A1 (HMGA1) is a chromatin-associated protein that regulates transcription and drives cancer progression. In this pan-cancer study, we analyzed multi-omics data to comprehensively characterize HMGA1’s expression patterns, prognostic significance, epigenetic regulation, and immunotherapy roles. We found that HMGA1 was markedly upregulated in most cancers, mainly driven by promoter hypomethylation and copy number alterations. Elevated HMGA1 expression was consistently associated with unfavorable patient survival, stemness features, and the activation of oncogenic signaling pathways. Crucially, HMGA1 expression correlated with an immune-excluded tumor microenvironment, characterized by suppressed stromal and immune scores. Even in tumors with immune infiltration, high HMGA1 predicted poor prognosis, likely mediated by enhanced regulatory T-cell (Treg) recruitment and impaired effector immune function. Moreover, HMGA1 levels were positively correlated with tumor mutational burden (TMB), and microsatellite instability (MSI), and immunotherapy-related checkpoints including PD-1, CTLA-4, and TIGIT. Drug sensitivity analysis further revealed that HMGA1 predicted resistance to AKT inhibitors, which was experimentally validated in breast cancer cells treated with Capivasertib. Collectively, our findings establish HMGA1 as a pivotal oncogenic regulator and a promising biomarker for prognosis and for guiding strategies in immunotherapy and overcoming targeted therapy resistance.

## Simple Summary

Cancer remains a leading cause of death worldwide, often because tumors can evade the immune system and become resistant to treatments. In this study, we investigated a protein called HMGA1, which is known to be present in high amounts in many cancers but whose broader role was not fully understood. By analyzing data from thousands of patients across numerous cancer types, we discovered that HMGA1 is frequently overactive due to specific chemical changes on its DNA. High levels of this protein were linked to poorer patient survival. We found that HMGA1 helps tumors hide from the body’s immune defenses and makes them resistant to a common class of drugs known as AKT inhibitors. We confirmed this drug resistance in breast cancer cells. Our work identifies HMGA1 as a key driver of cancer aggression and suggests that measuring its levels could help predict patient outcomes and guide more effective treatment strategies, particularly for immunotherapy.

## 1. Introduction

Cancer continues to pose a significant global health burden, with approximately 20 million new cases and 9.7 million deaths reported in 2022 [1]. Its rising incidence, driven by aging populations, environmental exposure and lifestyle changes, makes it one of the leading causes of morbidity and mortality worldwide [2,3,4,5]. Despite advances in early detection and therapeutic strategies, patient survival is often limited due to metastasis, therapy resistance, and tumor immune evasion [6,7]. The availability of large-scale genomic resources such as The Cancer Genome Atlas (TCGA) and complementary public databases has enabled systematic pan-cancer evaluations of oncogenes, facilitating biomarker discovery and the identification of novel therapeutic targets [8,9,10].

High mobility group (HMG) proteins are architectural chromatin regulators that modulate transcription, DNA repair, and nucleosome dynamics [11,12,13]. Among them, the High Mobility Group AT-Hook 1 (HMGA1) gene encodes a non-histone nuclear factor that binds AT-rich DNA regions, altering chromatin conformation and enhancing the accessibility of transcriptional machinery [14,15,16]. HMGA1 is normally expressed at low levels in adult tissues but is markedly upregulated in embryogenesis and malignancies, reflecting its role in cellular plasticity and transformation [17,18].

Furthermore, the dysregulation of HMGA1 is not merely a downstream consequence of transformation but also an active driver of tumorigenesis [19], shaped by distinct epigenetic mechanisms. These include promoter hypomethylation, which enhances transcription, as well as other layers of regulation such as changes in chromatin accessibility and specific histone modifications (e.g., H3K27ac and H3K4me3). Although post-translational modifications such as acetylation and phosphorylation have been shown to modulate HMGA1’s activity [20], these regulatory layers have also not been systematically investigated across cancer types. However, even for the fundamental mechanisms of promoter hypomethylation, a comprehensive, pan-cancer analysis is lacking. This gap underscores the need for our study, which focuses specifically on defining the role of DNA hypomethylation in driving HMGA1 overexpression across human cancers.

Accumulating evidence implicates HMGA1 as a potent oncogenic driver. Overexpression of HMGA1 promotes proliferation, epithelial–mesenchymal transition, and metastatic progression in breast, lung, and liver cancers [21,22,23,24]. Furthermore, HMGA1 has been linked to resistance against chemotherapeutics like kinase inhibitors through constitutive activation of the PI3K/AKT survival pathway [25,26,27]. This provides a strong rationale to investigate whether HMGA1 modulates sensitivity to Capivasertib (AZD5363), a clinically relevant AKT inhibitor recently approved for breast cancer therapy [28,29,30,31].

While prior studies have established HMGA1’s pro-tumorigenic functions within specific cancer types [32], the field still lacks a unified investigation that defines its overarching role across human malignancies. Most existing research is highly siloed, focused on single mechanisms, pathways or tumor types, which has limited broader insight into HMGA1 as a multifunctional oncoprotein. To address this gap, our study provides the first systematic pan-cancer analysis that simultaneously interrogates the genetic, transcriptonal, epigenetic, and immunologic dimensions of HMGA1. This integrative framework offers a level of mechanistic breadth and cross-cancer comparison that has not been explored previously.

The uniqueness of our approach lies in a multi-tiered design that integrates computational discovery with experimental validation. First, we developed a comprehensive computational framework to simultaneously analyze: (i) the genetic and epigenetic determinants of HMGA1 dysregulation; (ii) its impact on tumor immunity and patient prognosis; and (iii) its potential as a predictive biomarker for therapy response. To complement these bulk transcriptomic analyses and gain cellular-level insights, we leveraged single-cell RNA sequencing (scRNA-seq) data from the TISCH database, validating HMGA1 expression patterns across tumor-infiltrating immune and stromal cells populations. Second, and most importantly, our study bridges bioinformatic predictions with direct functional experiments. This confirms that key computational findings particularly HMGA1 mediated resistance to AKT inhibitions are rigorously tested and validated experimentally, a step often lacking in purely analytical studies.

Together, our findings establish HMGA1 as a central, multifunctional oncoprotein and a promising biomarker to guide immunotherapy and overcome treatment resistance in aggressive malignancies.

## 2. Materials and Methods

### 2.1. Data Acquisition and Preprocessing

Transcriptomic data (FPKM) and clinical information for 33 cancer types were obtained from The Cancer Genome Atlas (TCGA) via the University of California, Santa Cruz (UCSC Xena) platform (https://xena.ucsc.edu/ (accessed on 15 August 2025)) [33]. Normal tissues expression data were incorporated from the Genotype-Tissue Expression (GTEx) project (https://www.gtexportal.org/home/ (accessed on 15 August 2025)) [34]. All the expression values were log_2_(x + 0.001) transformed. Differential HMGA1 expression between tumor and normal tissues was further validated using the TIMER2.0 (https://compbio.cn/timer2/ (accessed on 20 August 2025)) [35]. Co-expression analysis between HMGA1 and other HMG family members were assessed on TCGA data and visualized using the R package corrplot (version 4.4.3).

### 2.2. Protein Expression, Cellular Localization and Prognostic Analysis

HMGA1 protein levels in ten matched tumor-normal pairs (e.g., BRCA, COAD, LIHC) were analyzed using Clinical Proteomic Tumor Analysis Consortium (CPTAC) data through the UALCAN portal (https://ualcan.path.uab.edu/ (accessed on 25 August 2025)) [36]. Subcellular localization was determined from immunofluorescence images in the Human Protein Atlas (HPA) (https://www.proteinatlas.org/ (accessed on 28 August 2025) [37], using antibody HPA065612 (Atlas Antibodies AB, Sigma-Aldrich, Saint Louis, MO, USA) in A-431, U2OS and PODO/TERT256 cell lines. Thus, the association between HMGA1 expression and patient survival was evaluated using the standardized TCGA TARGET GTEx (PANCAN) dataset with clinical follow-up from the TCGA Pan-Cancer Clinical Data Resource (https://gdc.cancer.gov/about-data/publications/PanCan-Clinical-2018 (accessed on 1 September 2025)) [38]. Samples with follow-up <30 days or from cancer types fewer than 10 samples were excluded. Univariate Cox proportional hazards models (R survival package (version 4.4.3)) were used to assess the impact on overall survival (OS) and progression-free interval (PFI).

### 2.3. Correlation Between HMGA1 Expression and DNA Methylation

We analyzed the relationship between HMGA1 expression and promoter DNA methylation using the Methsurv (https://biit.cs.ut.ee/methsurv/ (accessed on 5 September 2025)) [39] and the Shiny Methylation Analysis Resource (SMART) (http://www.bioinfo-zs.com/smartapp/ (accessed on 5 September 2025)) [40] tools. Seven promoter-associated CpG probes were identified, and their aggregated methylation levels were compared between tumor and normal tissues. Correlation between probe-specific methylation (e.g., cg00544436) and HMGA1 expression was assessed. Chromatin accessibility at the HMGA1 locus was investigated using DNase-seq data from the ENCODE database (https://www.encodeproject.org/ (accessed on 8 September 2025) [41], visualized with the integrative genomics viewer (IGV_win_2.8.10) [42]. The relationship between HMGA1 expression and copy number variation (CNV) status, derived from GISTIC2 data [43], was tested. For epitranscriptomic regulation, we evaluated the correlation between HMGA1 expression and 44 RNA modification regulators (10m1A, 13m5C, 21m6A) across TCGA cancer types.

### 2.4. Analysis of Stemness, Tumor Microenvironment, and Immune Infiltration

Stemness indices (DNAss, RNAss, EREG-METHss, and EREG-EXPss) from Malta et al. (Cell, 2018) [44] were correlated with HMGA1 expression. The tumor immune microenvironment was characterized using immune, stromal and ESTIMATE scores computed using the ESTIMATE package (version 1.0.13) [45]. To deepen the immune profiling, immune cell infiltration for six major lineages were estimated via TIMER2.0 (accessed on 12 September 2025), while 22 immune-cell subsets were quantified using CIBERSORT (https://cibersortx.stanford.edu/ (accessed on 13 September 2025)) [46] via IOBR (version 0.99.9) package. TMB was calculated from TCGA somatic mutation data using maftools (version 2.8.05) [47]. MSI scores were obtained from a published pan-cancer resource [48]. Finally, to validate and visualize these patterns at a cellular resolution, single-cell RNA sequencing data for six cancer types (e.g., BRCA, Glioma, LIHC, BLCA) were obtained from the Tumor Immune Single-Cell Hub (TISCH) database (http://tisch.comp-genomics.org/ (accessed on 15 September 2025)) [49]. HMGA1 expression patterns were visualized across annotated cell types using uniform manifold approximation and projection (UMAP) and violin plots.

### 2.5. Gene Set Enrichment and Drug Sensitivity Analysis

Gene enrichment analysis (GSEA) was performed using Broad Institute software (version 4.4.0) (https://www.gsea-msigdb.org/gsea/index.jsp (accessed on 18 September 2025)) [50,51], on the KEGG pathway collection [52], after stratifying samples by median HMGA1 expression. Protein–Protein interaction networks were generated with Gene-MANIA [53], and functional enrichment was assessed using Enricher (version 3.4) (https://maayanlab.cloud/Enrichr/ (accessed on 20 September 2025)) [53]. Drug sensitivity and basal expression profiles for cell lines were obtained from the GDSC portal (https://www.cancerrxgene.org/ (accessed on 25 September 2025)). For each drug, we tested the correlation between HMGA1 expression and the natural-log IC_50_ (LN(IC_50_)), including only drugs tested in ≥10 cell lines.

### 2.6. Evaluation of HMGA1 Expression and Functional Validation in Breast Cancer Cell Lines

HMGA1 expression across breast cancer subtypes was profiled using the GOBO (Gene expression-based Outcome for Breast cancer online) tool (version 1.0.3) (https://co.bmc.lu.se/gobo/ (accessed on 1 October 2025) [54], and validated with cell line data from UCSC Xena. For in vitro studies, MDA-MB-231, BT549, and T47D cells (American Type Culture Collection—ATCC, Manassas, VA, USA) were treated with the AKT inhibitor AZD5363 (MedChemExpress, Monmouth Junction, NJ, USA). HMGA1 transcript levels were measured by qRT-PCR using a QuantStudio 5 Real-Time PCR System (Thermo Fisher Scientific, Waltham, MA, USA), and SYBR Green Master Mix (Roche Diagnostics, Basel, Switzerland), cell viability was assessed using CCK-8 assays (Dojindo Molecular Technologies, Rockville, MD, USA), and protein expression was analyzed by Western blot using a ChemiDoc MP Imaging System (Bio-Red Laboratories, Hercules, CA, USA).

### 2.7. Statistical Analysis

Correlation analyses were performed using Pearson correlation. For bioinformatic analyses, significance was defined as *p* < 0.05 with false discovery rate (FDR) adjustment for multiple comparisons where applicable. For in vitro experiments, comparisons were made using two-way ANOVA test in GraphPad Prism (version 10.0.3; GraphPad Software, Boston, MA, USA), with *p* < 0.05 considered significant.

## 3. Results

### 3.1. HMGA1 Exhibits Pan-Cancer Overexpression and Nuclear Localization

**Figure 1:**
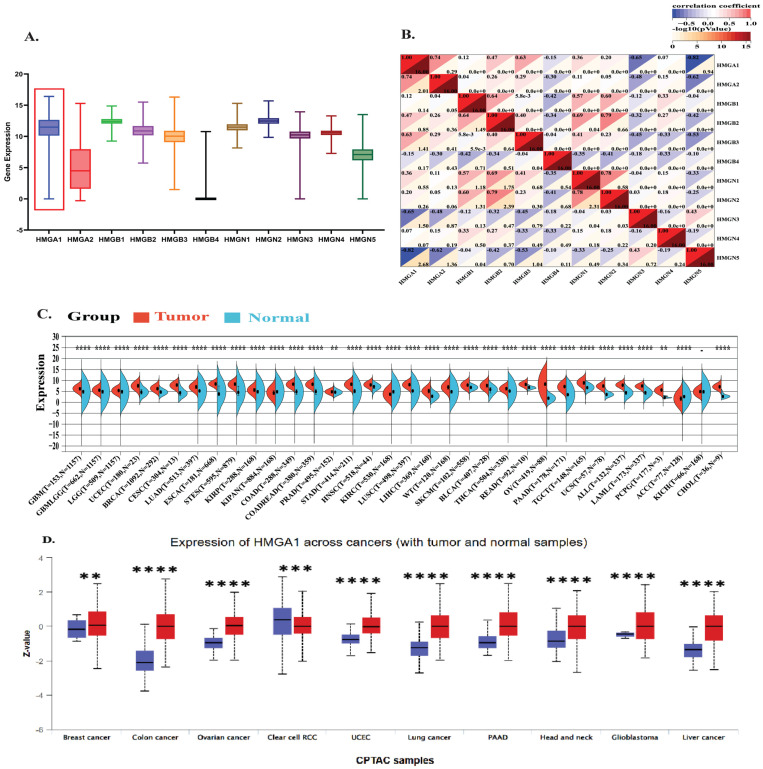
Pan−Cancer Overexpression and Clinical Significance of HMGA1 (**A**) Comparative expression analysis of HMG family genes across 33 TCGA tumor types. The red box highlights the HMGA1 cluster. (**B**) Co−Expression network and correlation coefficient among HMG family members across the TCGA Cohort (**C**) HMGA1 mRNA expression (log_2_ [x + 0.001]) analysis in tumors versus normal tissues based on integrated TCGA and GTEx datasets. (**D**) HMGA1 protein expression levels in 10 cancer types based on CPTAC datasets from the UALCAN portal. Red color represents tumor samples; blue represents normal tissue samples. Statistical Significance was determined by the unpaired Wilcoxon rank-sum test (** *p* < 0.01, *** *p* < 0.001, **** *p* < 0.0001).

Pan-cancer profiling showed that HMGA1 is broadly overexpressed and clinically relevant across multiple tumor types. Analysis of the HMGA family transcriptional landscape across 33 TCGA cancers revealed that HMGA1 displayed the highest median expression and broadest distribution among all HMG members (Figure 1A). Co-expression analysis showed a strong positive correlation with HMGA2 (r = 0.74, *p* < 0.001) and moderate correlations with HMGB2/3, while negative correlations with HMGN3/5, indicating a distinct oncogenic signature (Figure 1B).

Differential expression analyses confirmed significant HMGA1 upregulation in most tumors versus normal tissues, with the most notable increases in BRCA, LUAD, LIHC, and COAD (Supplementary Figure S1). Analysis of the standardized TCGA + GTEx pan-cancer datasets integration further reinforced these findings, with significant overexpression in GBM, LGG, BRCA, LUAD, COAD, LIHC, and PAAD (all *p* < 1 × 10^−8^) (Figure 1C). At the protein level, CPTAC dataset via UALCAN also confirmed HMGA1 overexpression in BRCA, COAD, OV, and other cancers (all *p* < 0.01). A notable exception was ccRCC, where HMGA1 was higher in normal tissue, suggesting tissue-specific regulation (Figure 1D). Immunofluorescence data from the Human Protein Atlas consistently showed predominant nuclear localization across cell lines, supporting its role as a chromatin-associated factor (Supplementary Figure S2).

### 3.2. High HMGA1 Expression Predicts Poor Prognosis

**Figure 2:**
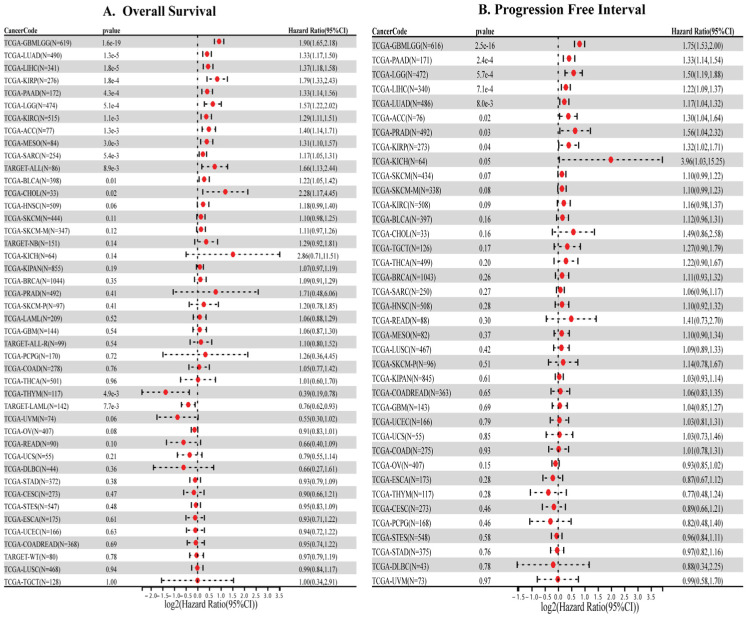
Prognostic significance of HMGA1 across cancers. (**A**) Overall survival (**B**) Progression free interval (PFI) in cancers.

Clinically, high HMGA1 expression was strongly associated with unfavorable outcomes. Elevated HMGA1 levels correlated with reduced overall survival (OS) in GBMLGG, LUAD, LIHC, KIRP, PAAD, and additional cancers (Figure 2A). Similarly, high HMGA1 predicted shorter progression-free interval (PFI) in GBMLGG, LUAD, LIHC, and PAAD (Figure 2B). Together, these results indicate that HMGA1 is a consistently overexpressed oncoprotein and a robust prognostic biomarker across diverse malignancies (Supplementary Table S1).

### 3.3. HMGA1 Overexpression Is Driven by Promoter Hypomethylation and Genetic Alterations

**Figure 3:**
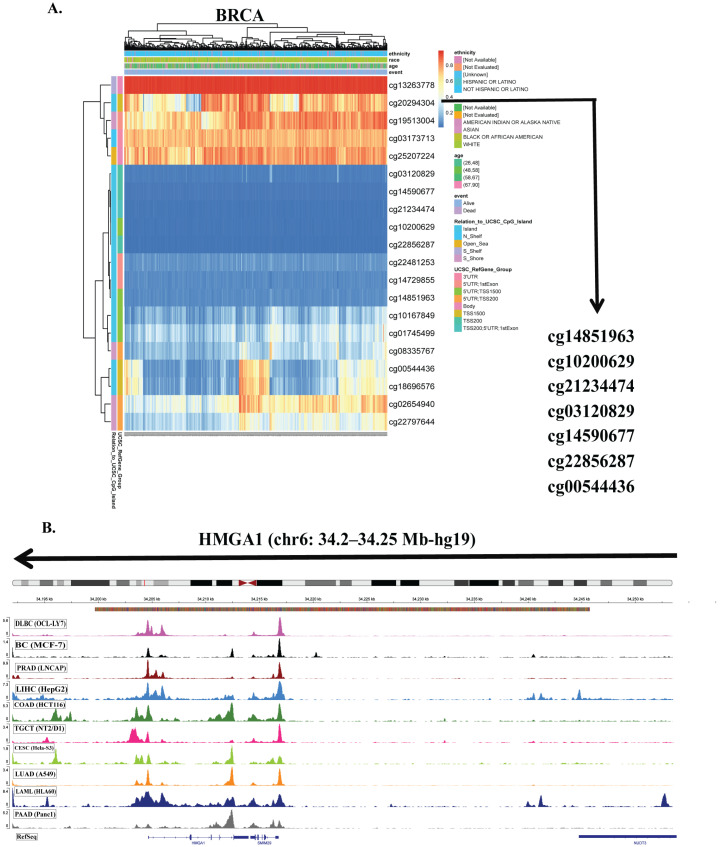
DNA methylation analysis of HMGA1 promoter regions. (**A**) Distribution of DNA methylation levels at the HMGA1 locus in the representative breast cancer (BRCA) samples. (**B**) DNase-seq profiles across 10 cancer types obtained from ENCODE database and visualized using the integrative genomic viewer (IGV).

**Figure 4:**
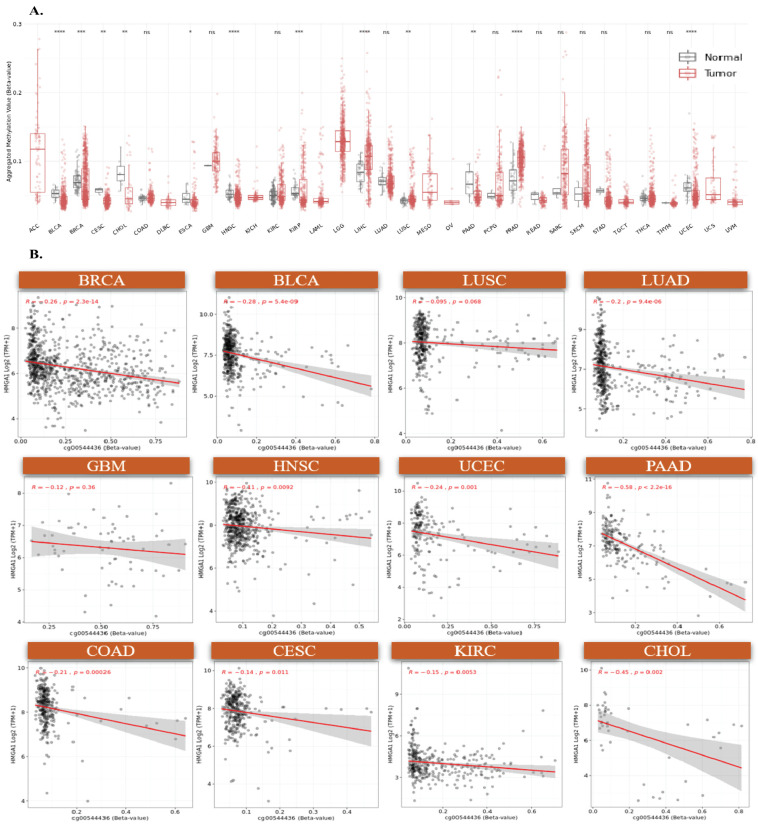
DNA methylation analysis of HMGA1 Island promoter regions. (**A**) Comparative analysis of aggregated methylation levels from seven HMGA1-associated probes across multiple cancers, (* *p* < 0.05, ** *p* < 0.01, *** *p* < 0.001, **** *p* < 0.001). (**B**) Correlation between DNA methylation at probe cg00544436 and HMGA1 expression in twelve tumor types. Colored dots represent individual samples for each tumor type (as labeled), and the gray shadow indicates the 95% confidence interval of the fitted trend line. ns indicates no significant difference.

To identify mechanisms underlying HMGA1 upregulation in cancer, we first examined promoter methylation across TCGA tumors. Seven CpG sites located within the promoter-associated regions (TSS200, TSS1500, and 5’UTR) were consistently hypomethylated in tumors with elevated HMGA1 expression (Figure 3A, Supplementary Figure S3). Consistent with an epigenetically permissive promoter, DNase-seq profiles from ENCODE showed increased chromatin accessibility at the HMGA1 promoter across ten cancer types (Figure 3B), supporting a model in which promoter hypomethylation facilitates transcriptional activation. Aggregated methylation of the seven probes (SMART App) was significantly reduced in multiple tumors, including BLCA, BRCA, CESC, HNSC, KIRP, PAAD, and UCEC, whereas no significant changes were observed in THYM, THCA, TGCT, READ, SARC, and STAD (Figure 4A). Together, these results indicate that promoter hypomethylation underlies HMGA1 activation in a subset of cancers. Because individual CpG sites can have differential regulatory importance, we examined probe-level associations. The TSS-proximal probe cg00544436 showed the strongest negative correlations with HMGA1 expression in BRCA (r = −0.26, *p* < 2.3 × 10^−14^), BLCA (r = −0.28, *p* < 5.4 × 10^−9^), LUAD (r = −0.2, *p* < 9.4 × 10^−6^) and PAAD (r = −0.58, *p* < 2.2 × 10^−16^), highlighting its regulatory importance (Figure 4B). These data suggest that promoter hypomethylation, particularly at transcriptionally sensitive CpG sites, is a key driver of HMGA1 overexpression.

To determine whether additional mechanisms to dysregulation, we evaluated more genomic and epitranscriptomic features through Copy number variation (CNV) analysis that revealed HMGA1 amplification occurs across several cancers and is strongly associated with increased expression (Supplementary Figure S4A). Furthermore, HMGA1 expression showed pervasive positive correlations with regulators of major RNA modification pathways (m6A, m5C, m1A), including METTL3, ALYREF, TRMT6 (Supplementary Figure S4B). These associations suggest that epitranscriptomic programs may reinforce HMGA1 overexpression or stabilize HMGA1 transcripts in tumors. Collectively, these findings indicate that HMGA1 dysregulation arises from multiple convergent mechanisms, including promoter hypomethylation, enhanced chromatin accessibility, CNV amplification, and interaction with RNA modification pathways.

### 3.4. HMGA1 Expression Correlates with Tumor Stemness and Immune Exclusion

**Figure 5:**
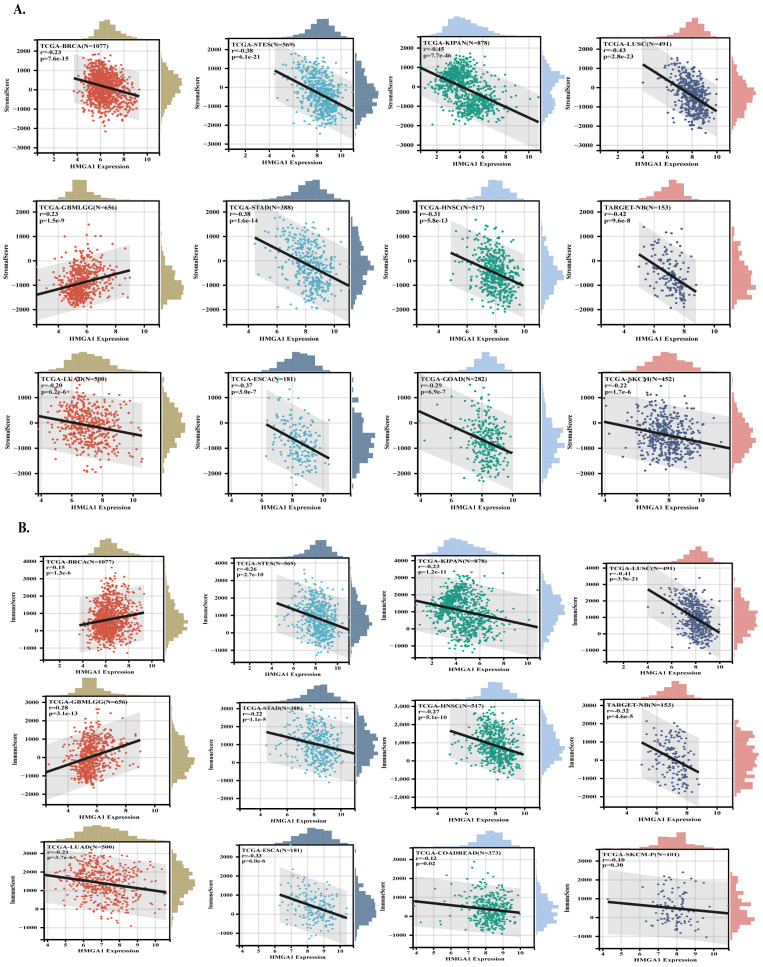
HMGA1 links to tumor stemness and immune exclusion across cancers. (**A**,**B**) Relationships between HMGA1 expression and ESTIMATE−derived immune and stromal scores. Colored dots represent individual samples for each tumor type (as labeled), and the gray shadow indicates the 95% confidence interval of the fitted trend line.

HMGA1 expression showed a strong positive correlation with stemness indices derived from RNA expression (RNAss) and DNA methylation (DNAss) in the majority of tumor types, particularly BRCA, LUAD, and STAD, indicating its association with de-differentiated tumor states. In contrast, a few cancer types such as KIPAN showed inverse correlations, suggesting context-dependent effects (Supplementary Figure S5). To evaluate how HMGA1 relates to the tumor microenvironment (TME), we examined ESTIMATE stromal and immune scores. HMGA1 was broadly associated with reduced stromal and immune scores in multiple cancers, consistent with an immune-excluded phenotype. However, several tumor types with positive correlations (e.g., GBMLGG, LGG, and SARC) are known to harbor immunosuppressive infiltrates, suggesting that HMGA1 may be linked to dysfunctional rather than effective immune presence. The strongest associations were observed in KIPAN, LUSC, STES, BRCA, STAD, HNSC, GBMLGG, NB, ESCA, COAD, SKCM and LUAD for stromal scores, and LUSC, GBMLGG, KIPAN, STES, HNSC, KIRC, SARC, PRAD, THCA, BRCA, LGG and LUAD for immune scores (Figure 5A,B).

### 3.5. HMGA1 Expression Fosters an Immunosuppressive Tumor Microenvironment Across Multiple Cancers

**Figure 6:**
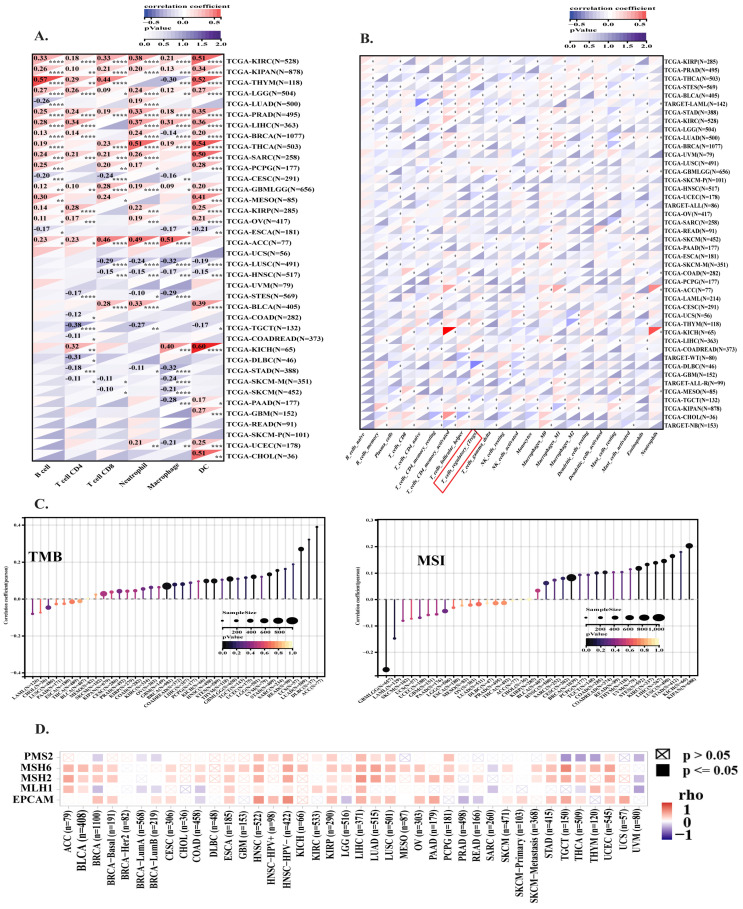
Associations of HMGA1 with immune infiltration, TMB, and MSI across cancers. (**A**) Correlation of HMGA1 with six major immune cell types across 38 cancers. (**B**) Heatmap of HMGA1 and 22 immune-cell subsets represented upregulation of T-regulatory cells among different cancers. (**C**) HMGA1 expression positively correlated with TMB and MSI in several cancers. (**D**) Correlations between HMGA1 and mismatch repair genes (EPCAM, MSH2, MSH6, and PMS2) across cancers. Statistical Significance is indicated as follows: (* *p* < 0.05, ** *p* < 0.01, *** *p* < 0.001, **** *p* < 0.001).

Analysis of 36 cancer types revealed that HMGA1 expression significantly correlates with altered infiltration of various immune cells, including B cells, T cells, macrophages, neutrophils, and dendritic cells (Figure 6A). A strong negative correlation with macrophage infiltration was observed in cancers such as BRCA, and THYM. Notably, HMGA1 showed a consistent positive association with the activation of regulatory T cells (Tregs) in several cancers, including BRCA, GBM, and LIHC (Figure 6B). This pattern was supported by the concurrent upregulation of inhibitory checkpoints (e.g., CTLA-4, PD-1, TIGIT), and Treg-related markers (e.g., CD39, CD35), suggesting HMGA1 contributes to an immunosuppressive TME. In contrast, immune stimulatory markers like CD28 and TNFRSF6 (4-1BB) were negatively correlated with HMGA1 (Supplementary Figure S6).

Further supporting this, the expression of the Treg-recruiting chemokine axis (CCR4/CCR8 and their ligands CCL17/CCL22) was positively correlated with HMGA1. Conversely, the CXCR3/CXCL9/CXCL10 axis, which attracts effector T cells, was negatively correlated. MHC Class I expression, critical for CD8+ T cell activation, was also negatively correlated with HMGA1, suggesting a mechanism for impaired antigen presentation and immune evasion (Supplementary Figure S7). To explore the molecular mechanisms, we analyzed the relationship between HMGA1 and genomic instability. HMGA1 expression was positively correlated with both tumor mutational burden (TMB) and microsatellite instability (MSI) in numerous cancers (Figure 6C). Since MSI is often driven by DNA mismatch repair (MMR) defects, we analyzed key MMR genes and found their expression was positively correlated with HMGA1 in most tumors (Figure 6D), indicating a potential link between HMGA1 and DNA repair mechanisms.

To strengthen the bulk transcriptomic findings at cellular resolution and establish the pan-cancer relevance of HMGA1-driven immune evasion, we performed comprehensive single-cell RNA sequencing analysis across six malignancies (Supplementary Table S2). This high-resolution approach revealed that HMGA1 exhibits conserved expression patterns within tumor immune microenvironments, with particularly strong expression in immunosuppressive cell populations. In BRCA, HMGA1 demonstrated prominent expression across multiple T lymphocyte populations, including CD4+ conventional T cells, CD8+ T cells, and regulatory T cells (Treg) (Supplementary Figure S8A). This lymphoid-focused pattern was conserved in glioma, LIHC, and CRC, with consistent HMGA1 detection in Tregs and exhausted CD8+ T cell populations (Supplementary Figure S8B–D). Notably, ESCA displayed a distinct expression profile, with HMGA1 detected in malignant epithelial cells, cancer-associated fibroblasts, and CD8+ exhausted T cells (Supplementary Figure S8E). BLCA exhibited the most comprehensive HMGA1 distribution, spanning Tregs, natural killer cells, and multiple T cell subsets (Supplementary Figure S8F). Crucially, quantitative analysis confirmed that all six cancer types (Supplementary Table S3), Tregs consistently maintained HMGA1 expression exceeding log-normalized level of 2, representing one of the most reliably high-expressing immune populations. While the absolute highest-expressing cell type varied by cancer context, this conserved high expression in Tregs provide cellular-level verification that HMGA1’s might also play a role in fostering immunosuppressive microenvironments and establishes its conserved function as an immune modulator across diverse malignancies.

### 3.6. Gene Set Enrichment and Interaction Analysis of HMGA1 Across Cancers

**Figure 7:**
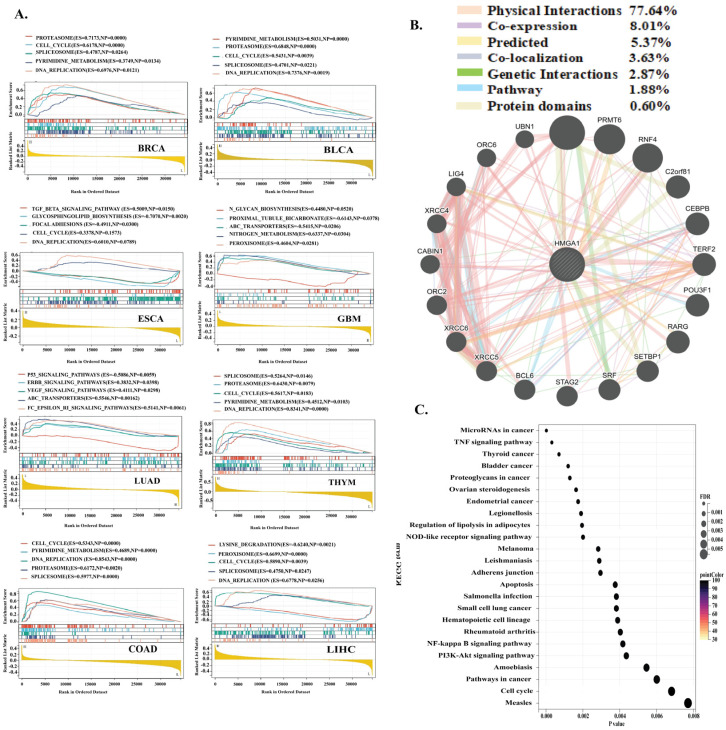
HMGA1 Drives Conserved and Context-Specific Oncogenic Programs. (**A**) GSEA reveals universal proliferative pathways and tissue−specific alterations. (**B**) Interaction network positions HMGA1 as a chromatin regulatory hub. (**C**) GO analysis confirms roles in cell division and DNA metabolism.

To define the oncogenic pathways driven by HMGA1, we performed Gene Set Enrichment Analysis (GSEA) across eight cancer types. This revealed that HMGA1 consistently activates a core set of pathways essential for rapid cell proliferation, including the cell cycle, DNA replication, pyrimidine metabolism, spliceosome, and proteasome, which were enriched in cancers such as BRCA, COAD, and LIHC. Beyond this universal signature, HMGA1 exhibited tissue-specific pathway alterations. In LUAD, it was associated with activation of oncogenic ERBB signaling and suppression of p53, whereas in ESCA, HMGA1 negatively regulated TGF-β signaling and focal adhesion, suggesting a context-dependent role in promoting invasion (Figure 7A). Protein–Protein interaction analysis positioned HMGA1 as a central hub linked to partners such as HMGA2 and PRMT6, primarily through physical and co-expression links (Figure 7B). Complementary Gene Ontology (GO) analysis further reinforced its involvement in mitotic processes, DNA repair, and microtubule indents (Figure 7C). Collectively these findings indicate that HMGA1 orchestrates a versatile oncogenic program, utilizing common proliferation and metabolic programs across cancers while modulating context-specific pathways to support tumor survival and progression.

### 3.7. HMGA1 Associates with Drug-Specific Sensitivity Patterns

**Figure 8:**
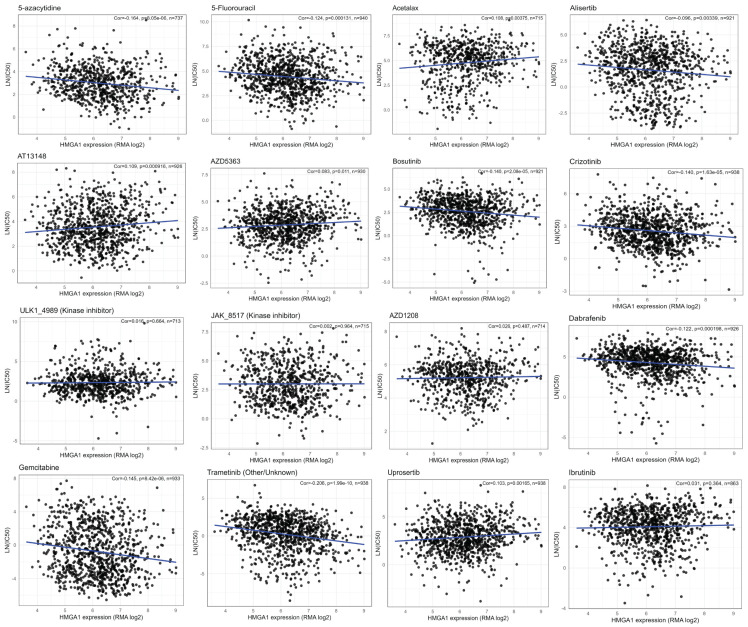
HMGA1 drug response across GDSC cell lines shows HMGA1 expression (RMA log) versus LN(IC50) across GDSC cell lines. Each black circle represents an individual cell line, and the blue line indicates the fitted least-squares regression trend. Insets report the Pearson correlation coefficient (r), two-sided *p*−value, and sample size (n). Higher LN(IC_50_) values indicate lower drug sensitivity.

Across GDSC cell lines, HMGA1 expression showed two distinct correlation patterns with drug responses. Negative correlations (higher HMGA1 = lower LN (IC50), indicating greater sensitivity) were observed for several cytotoxic and targeted agents, including 5-azacytidine, 5-fluorouracil, gemcitabine, dabrafenib (BRAF inhibitor), crizotinib (ALK/MET inhibitor), alisertib (AURKA inhibitor), and bosutinib (SRC/ABL/SFK inhibitor). In contrast, positive correlations (higher HMGA1 = higher LN (IC50), indicating reduced sensitivity) were found for AZD5363/capivasertib and uprosertib (AKT inhibitors) and well as acetalax, suggesting a HMGA1-linked resistance phenotype. Several drugs demonstrated minimal or no linear association with HMGA1 levels, including ibrutinib, ULK_4989, JAK_8517, GSK266616AC, AZD1208 (Figure 8). In summary, these results reveals a clinically meaningful dichotomy: HMGA1-high tumors may be more sensitive to DNA-targeting antimetabolites and inhibitors of BRAF, ALK/MET, AURKA and SRC-family Kinases, while HMGA1-high tumors may exhibit intrinsic resistance to AKT-targeted therapies. These patterns provide mechanistic hypotheses for future validation and suggest that HMGA1 may serve as a dual-direction predictive biomarker depending on drug class.

### 3.8. HMGA1 Expression Patterns and Differential Response to AZD5363 in Breast Cancer

**Figure 9:**
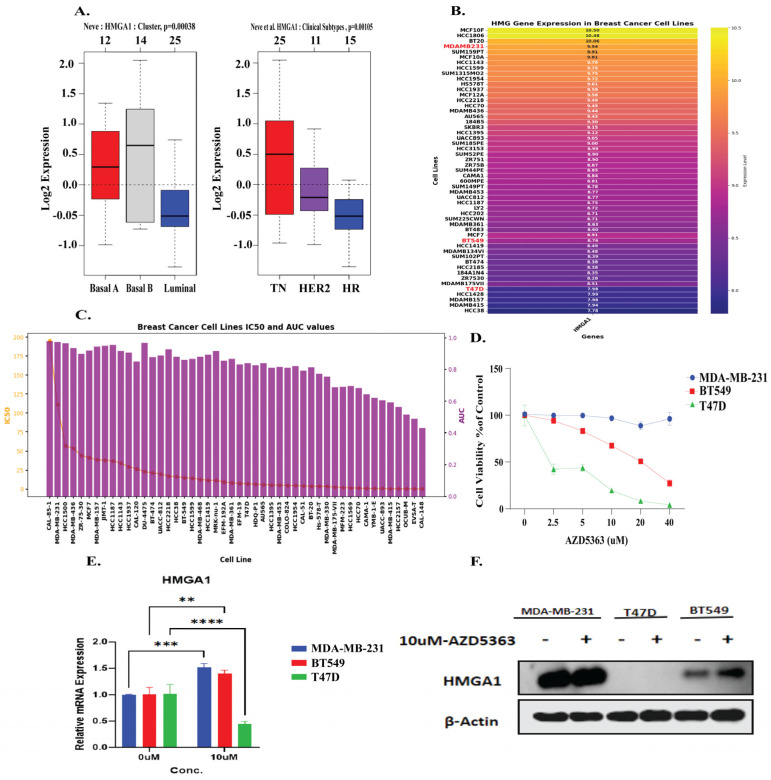
HMGA1 mediate resistance to the AKT inhibitor AZD5363 in breast cancer (**A**) HMGA1 expression is elevated in basal-like TNBC subtypes (GOBO). (**B**,**C**) HMGA1 is highly expressed in TNBC cell lines (UCSC Xena), and correlates with AZD5363 resistance (GDSC). (**D**) Viability assays confirm MDA-MB-231 and BT549 are resistant, while luminal T47D cells are sensitive. (**E**,**F**) AZD5363 treatment suppresses HMGA1 in sensitive T47D cells (qRT-PCR) but induces its expression in resistant TNBC cells (qRT-PCR and Western blot). Statistical level are represented as (** *p* < 0.01, *** *p* < 0.001, **** *p* < 0.001).

To validate the role of HMGA1 in cancer, we selected representative breast cancer cell lines to further verify HMGA1 role. GOBO tool showed that HMGA1 expression was significantly higher in basal-like and triple-negative breast cancer subtypes compared with luminal and HER2-positive tumors (*p* < 0.001) (Figure 9A). UCSC Xena database analysis demonstrated elevated HMGA1 mRNA levels in TNBC cell lines such as MDA-MB-231 and BT549 relative to luminal T47D cells (Figure 9B). Moreover, GDSC tool showed pharmacogenomic analysis of breast cancer cell lines revealed heterogeneous IC50 and AUC distributions for AZD5363 (Figure 9C). Consistent with these predictions, drug sensitivity assays revealed a stark contrast in response to the AKT inhibitor Capivasertib (AZD5363). The luminal T47D cell line, which exhibits low HMGA1 mRNA and undetectable HMGA1 protein, was highly sensitive to treatment. This sensitivity is consistent with the known reliance of luminal breast cancer subtype on the PI3K/AKT pathway [55,56]. In contrast, the triple-negative breast cancer (TNBC) cell lines MDA-MB-231 and BT549, which express high levels of both HMGA1 mRNA and protein, demonstrated significant resistance (Figure 9D). Notably, treatment with AZD5363 induced a feedback upregulation of HMGA1 mRNA in the resistant TNBC lines, while suppressing it in the sensitive T47D cells (Figure 9E). This differential regulation was confirmed at the protein level in TNBC cells, whereas HMGA1 protein was further elevated after post-treatment (Figure 9F). The absence of detectable HMGA1 protein in T47D cells despite the presence of its mRNA may be explained by post-transcriptional regulatory mechanisms, such as microRNA-mediated repression on rapid protein turnover, which are common in luminal subtypes [57,58]. Collectively, these findings underscore HMGA1 as a key marker and potential mediator of resistance to AKT inhibition in aggressive breast cancer subtypes.

## 4. Discussion

The dynamic interplay between chromatin remodeling, immune evasion, and therapy resistance represents a central challenge in oncology [59,60]. Our pan-cancer analysis establishes the architectural transcription factor HMGA1 as a critical node at the intersection of these processes. We demonstrate that HMGA1 is not only a ubiquitous marker of aggressive disease but also a key driver of a stem-like, immunosuppressive tumor microenvironment and a novel determinant of resistance to AKT inhibition.

HMGA1 is a chromatin-binding protein that alters nucleosome architecture and promotes transcriptional accessibility [61]. Under normal physiological conditions, HMGA1 expression is low in adult tissues, but it is strongly reactivated during embryogenesis and in malignant state. Previous studies have shown that HMGA1 supports oncogenic transformation, epithelial–mesenchymal transition, and metastasis in many cancers [62,63,64]. Our analysis confirmed that HMGA1 is broadly overexpressed in cancer tissues compared to matched normal controls and that this upregulation is mainly derived by promoter hypomethylation and copy number amplification. In agreement with earlier reports, HMGA1 overexpression correlated significantly with poor overall survival and progression-free interval in multiple cancers including GBMLGG, LUAD, LIHC, KIRP, and PAAD [65,66,67,68].

Beyond these established mechanisms, our analysis uncovered novel dimensions of HMGA1 regulation and its potential interplay with the epitranscriptome. HMGA1 overexpression was frequently driven by CNV amplification, reinforcing its role as a genomic oncogene under selective pressure. More intriguingly, we discovered that HMGA1 expression shows widespread positive correlations with regulators of key RNA modifications, including m6A, m5C, and m1A, across diverse cancer types. This suggest a potential co-regulatory network where HMGA1, as a master chromatin organizer [69], may influence the transcription of these modifiers, or conversely, where epitranscriptomic machinery may post-transcriptionally stabilize [70] HMGA1 mRNA or the transcripts of its target genes. This synergistic relationship between chromatin remodeling and RNA modification could represent a powerful, self-reinforcing circuit that amplifies oncogenic transcriptional programs and contributes to the pervasive influence of HMGA1 across cancer hallmarks.

A particular novel finding of our study is the robust association between HMGA1 and an immune-excluded tumor microenvironment. We observed that high HMGA1 expression was negatively associated with stromal and immune scores in many cancers, suggesting its involvement in generating an immune-excluded tumor microenvironment. Interestingly, in some cancer types where HMGA1 expression showed a positive association with immune scores, patients still exhibited poor prognosis. This observation implies that although immune cells may be present within tumor microenvironment, their anti-tumor functions could be impaired or suppressed potentially due to HMGA1-driven immunoregulatory mechanisms [71]. To directly validate this hypothesis at cellular resolution, single-cell RNA sequencing across six cancer types was performed. This analysis confirmed that HMGA1 is consistently highly expressed within key immunosuppressive compartments. Most notably, regulatory T cells (Tregs) maintained reliably high HMGA1 expression across diverse malignancies, including BRCA, LIHC, and CRC. This conserved enrichment provides a direct cellular-level explanation for the observed immune dysfunction: HMGA1 is not merely correlating with an immune-excluded state but is actively expressed within the different cells that drive immunosuppression. Furthermore, its frequent detection in exhausted CD8+T cell populations suggests a dual role in simultaneously impairing effector function and promoting regulatory suppression. While the specific cellular expression patterns showed context-dependence such as prominent expression in malignant cells and fibroblasts in ESCA the consistent association with immunosuppressive elements firmly establishes HMGA1 as a conserved immune modulator across cancers.

Our analysis also provides compelling insights into the potential mechanisms through which HMGA1 orchestrates this immunosuppressive landscape. As a master chromatin organizer, HMGA1 likely remodels the epigenetic landscape to favor the transcription of immunosuppressive programs [72,73]. This is supported by our findings that HMGA1 expression strongly correlates with: (1) Enhanced Treg recruitment through positive associations with chemokine receptors CCR4/CCR8 and their ligands CCL17/CCL22, creating a chemotactic gradient that attracts immunosuppressive Tregs to tumor site [74,75]; (2) Upregulation of multiple immune checkpoint molecules including PDCD1 (PD-1), CTLA4, TIGIT, and LAG3, potentially through HMGA1’s ability to enhance chromatin accessibility at these loci [76]; and (3) Impaired effector T-cell function through negative associations with MHC class 1 molecules and T-cell recruiting chemokines (CXCL9, CXCL10), effectively creating a cold tumor immune microenvironment [77,78]. The translational relevance of these findings is also substantial. It suggests that HMGA1 represents a promising therapeutic target to reverse immune exclusion and potentially sensitize tumors to immunotherapy [79,80]. Several targeting strategies could be explored: direct HMGA1 inhibition through small molecules that disrupt its DNA-binding capability [81]; combination approaches pairing HMGA1-targeting strategies with existing immune checkpoint inhibitors [82]; or targeting downstream effectors such as the CCL17/CCL22-CCR4 axis to disrupt Treg recruitment [83]. These approaches could be particularly relevant for HMGA1-high tumors that currently respond poorly to single-agent immunotherapy, offering a rational combination strategy to overcome resistance.

We also identified strong positive associations between HMGA1 expression and TMB and MSI in several cancer types, along with correlations with mismatch repair gene expression. Since TMB and MSI are widely recognized biomarkers for immunotherapy responsiveness [84], these findings suggest that HMGA1 could be integrated into predictive frameworks for immunotherapy outcomes. However, given HMGA1 clear links to immunosuppressive infiltration, its predictive role may be complex and context-dependent. Enrichment analysis further indicated that HMGA1 regulates pathways central to tumor cell proliferation and survival, including cell cycle progression, DNA replication, proteasome activity, and pyrimidine metabolism. In LUAD and ESCA, additional enrichment of ERBB signaling, ABC transporters, and suppression of p53 signaling was observed, pointing toward tumor-specific effects.

Our findings that HMGA1 expression predicts resistance to AKT inhibitors, including Capivasertib, provide a direct functional explanation for the poor prognosis associated with HMGA1-high tumors. While prior studies suggested HMGA1 could activate the PI3K/AKT survival pathway [85], we now demonstrate that this translates into clinically relevant resistance to a therapeutic AKT inhibitors. Crucially, the feedback upregulation of HMGA1 in resistant TNBC cells upon drug exposure reveals a dynamic, self-reinforcing loop that may solidify the resistance phenotype. This aligns with and extends previous models of HMGA1-driven drug resistance [86], firmly positioning it not only as a pathway activator but as a key mediator of adaptive resistance and a compelling biomarker for stratifying patients for AKT-targeted therapies.

While this study provides a comprehensive multi-omics perspective, several limitations should be acknowledged. First, most of the evidence presented here is correlative, and although the associations between HMGA1, immune exclusion, and drug response are strong and biologically coherent, they do not establish causality. Definitive mechanistic clarification will require experimental studies such as CRISPR/Cas9-mediated knockdown or overexpression, rescue assays to confirm pathway dependency, and in vivo tumor models to evaluate how HMGA1 influences immune cell dynamics within the tumor microenvironment. Second, the functional validation performed in breast cancer models provides proof-of-concept but may not fully reflect HMGA1 activity across diverse cancer types. Expanding validation efforts to additional tumor contexts and employing co-culture or immune-competent models will be essential to distinguish tumor-intrinsic effects from microenvironmental interactions. Despite these limitations, the consistency of results across independent datasets and pharmacogenomic platforms strongly supports HMGA1 as a clinically relevant regulatory hub and provides clear mechanistic directions for future investigation.

## 5. Conclusions

In summary, our comprehensive pan-cancer analysis defines HMGA1 as a master oncogenic regulator whose influence extends from genetic and epitranscriptomic regulation to the control of the tumor immune landscape. We demonstrate that its overexpression, driven by promoter hypomethylation, CNV, and a potential novel interplay with RNA modifiers, confers a poor prognosis. Functionally, HMGA1 orchestrates a multifaceted immunosuppressive program recruiting Tregs, upregulating immune checkpoints, and excluding effector T-cells which is validated by both bulk and single-cell transcriptomics. Crucially, we identify HMGA1 as a key determinant of resistance to AKT inhibitors, like Capivasertib, revealing a therapeutically targetable vulnerability. We thus propose HMGA1 not only as a robust prognostic biomarker but also a compelling therapeutic node, whose inhibition could simultaneously reverse immune exclusion and overcome targeted therapy resistance in aggressive malignancies.

## Data Availability

Data is contained within article and supplementary data files.

## Supplementary Materials

The following supporting information can be downloaded at https://www.mdpi.com/article/10.3390/biology14121758/s1. Figure S1. HMGA1 expression profiles across cancers. (A) Differential expression of HMGA1 between tumor and normal tissues using TIMER2.0. Figure S2. Protein expression and subcellular localization of HMGA1. (A) Immunofluorescence images from the Human Protein Atlas (HPA) showing HMGA1 localization in A-431, PODO/TERT256, and U2OS cell lines, with predominant localization in the nucleoplasm and nuclear membrane, and additional presence in the cytosol. Figure S3. DNA methylation analysis of HMGA1 promoter sites. (A–J) Distribution of DNA methylation at the HMGA1 locus in the representative cancers. Figure S4. Genetic Variations and Epitranscriptomic Correlations of HMGA1 (A) Boxplots show HMGA1 expression in samples classified as Neutral, Gain or loss across TCGA cancers. Statistical differences were determined by Wilcoxon or Kruskal-Wallis tests. *** *p* < 0.001; ** *p* < 0.01; * *p* < 0.05; ns, not significant. (B) Heatmap showing Pearson correlation coefficients between HMGA1 and RNA modification-related genes, including m6A, m5C, and m1A regulators (classified as writers, readers, or erasers). Red indicates positive and blue indicates negative correlation. Figure S5. (A,B) Correlations of HMGA1 expression with stemness indices (DNAss, RNAss, EREG-METHss, EREG-EXPSs) in representative cancers, showing strong positive associations. Figure S6. HMGA1 correlation with immune inhibitory checkpoints and Treg-related markers in cancers. Figure S7. Correlation of immune markers and chemokines with HMGA1 in cancers. Figure S8. Single-Cell expression landscape of HMGA1 across six cancer types (A–F) Single-cell RNA-sequencing data from TISCH demonstrate HMGA1 expression across major cellular compartments in Breast cancer (A), Glioma (B), Liver Hepatocarcinoma cancer (C), Colorectal cancer (D), Esophageal cancer (E), and Bladder cancer (F). Figure S9. RAW-WB-results. Table S1: Cancers types abbreviations. Table S2: Immune Infiltration Analysis (ESTIMATE). Table S3. sc−RNA Sequencing Datasets
