# Supplementary material for: Promoter Hypomethylation Unleashes HMGA1 to Orchestrate Immune Evasion and Therapy Resistance Across Cancers
Source: Biology (Basel). 2025 Dec 9;14(12):1758. doi: 10.3390/biology14121758 (PMC12730571; doi:10.3390/biology14121758)
Supplement: Supplementary file 1 [file biology-14-01758-s001.zip › Figure S9. RAW-WB-results.pdf]

(A)

Beta-Actin 42kDa

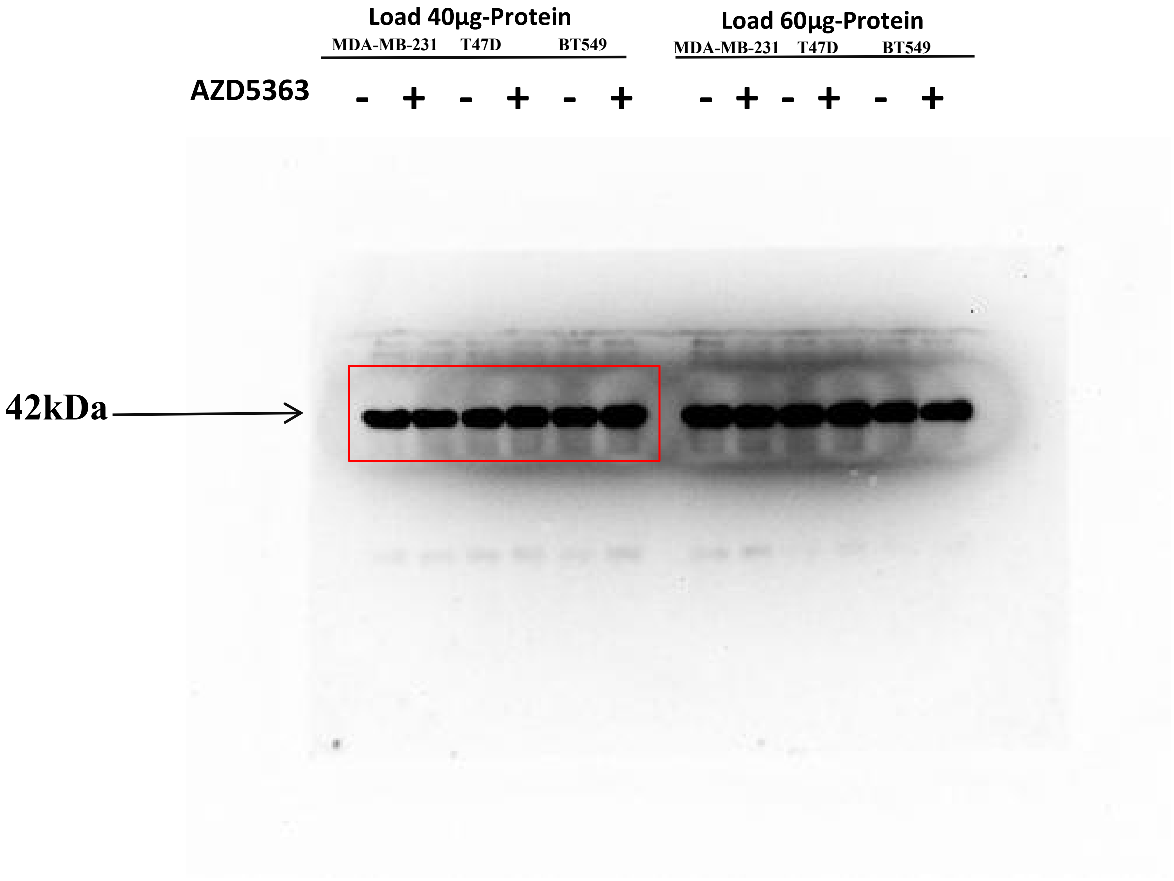

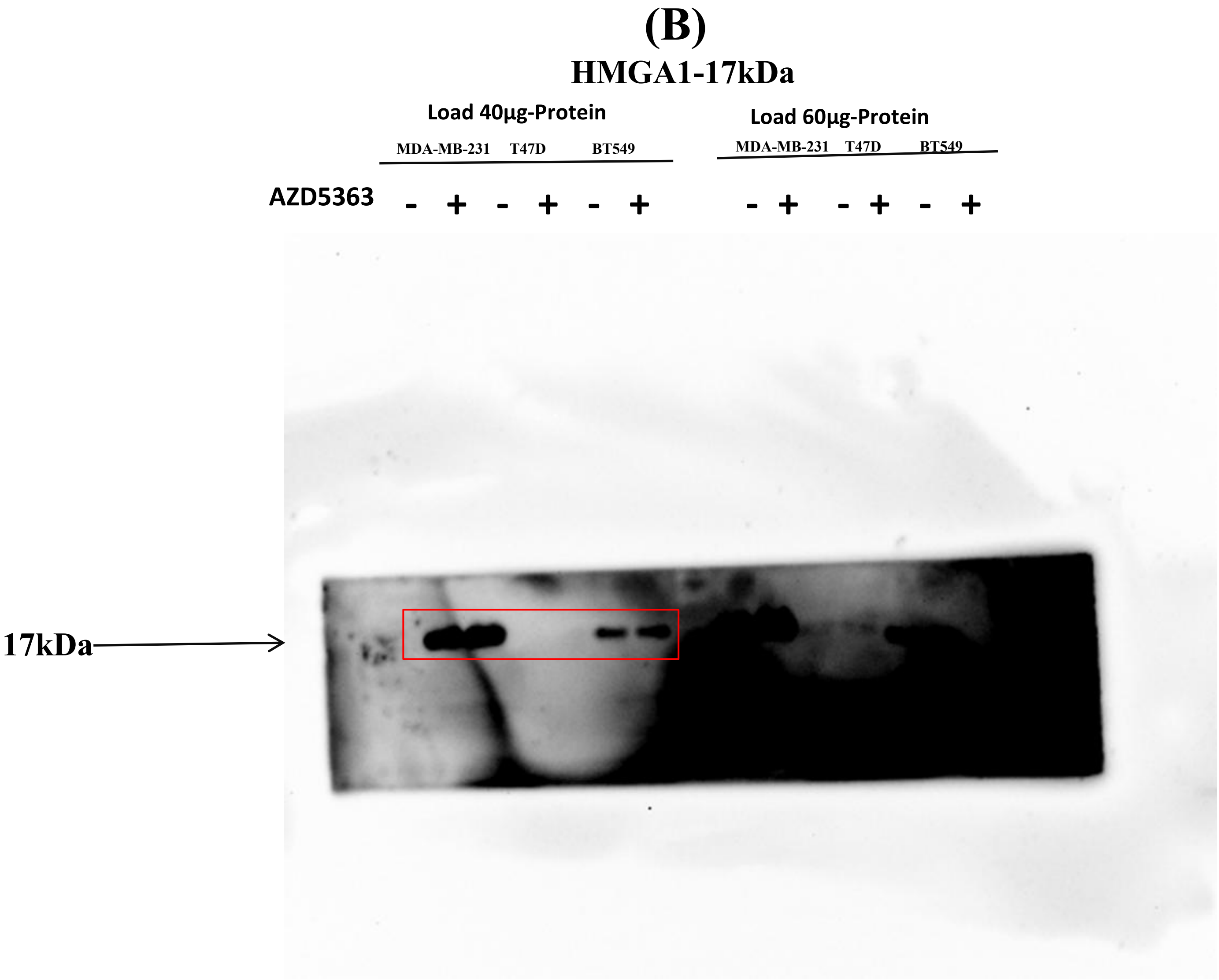

Figure S9. HMGA1 expression in breast cancer cell lines after AZD5363 treatment and corrosponding loading control.

Whole-cell lysates from MDA-MB-231, T47D, and BT549 cells treated with AZD5363 (+) or vehicle control (-) were separated by SDS- PAGE and analyzed by Immunoblotting. **(A)** β-actin (42kDa, arrow) was used as a loading control. **(B)** HMGA1 was detected at the expected molecular weight (17kDa arrow). Two total protein loading amounts were tested 40µg (left) and 60µg (right) per lane. Because the 60µg condition produced higher background and reduced clarity for the low-molecular-weight HMGA1 band, 40µg total protein was selected as the optimal loading amount for HMGA1 visualization and interpretation.
