# Supplementary material for: Promoter Hypomethylation Unleashes HMGA1 to Orchestrate Immune Evasion and Therapy Resistance Across Cancers
Source: Biology (Basel). 2025 Dec 9;14(12):1758. doi: 10.3390/biology14121758 (PMC12730571; doi:10.3390/biology14121758)
Supplement: Supplementary file 1 [file biology-14-01758-s001.zip › Supplementary data-Manuscript.pdf]

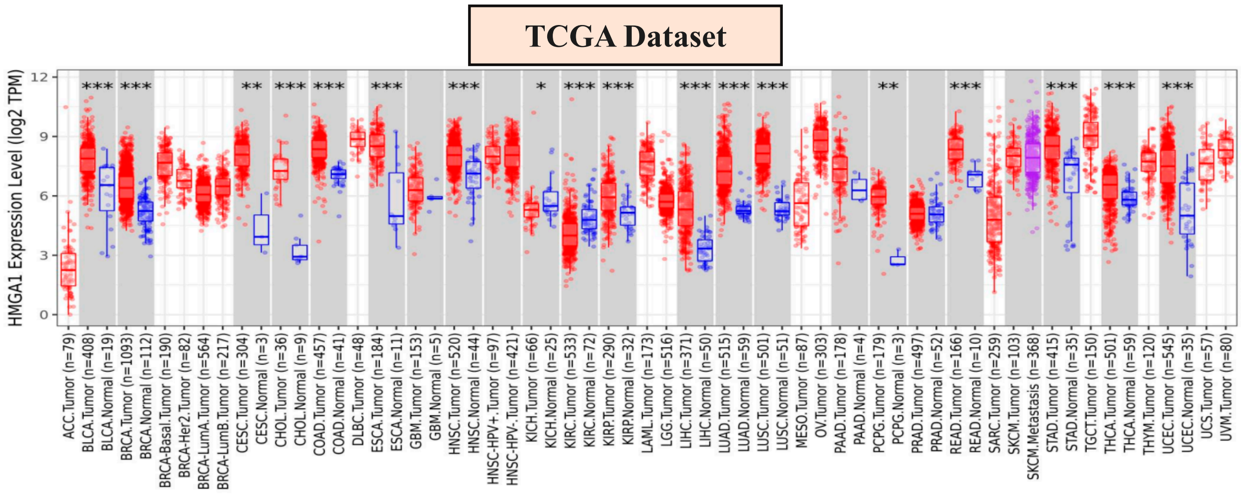

Supplementary Figure S1. HMGA1 expression profiles across cancers. **(A)** Differential expression of HMGA1 between tumor and normal tissues using TIMER2.0.

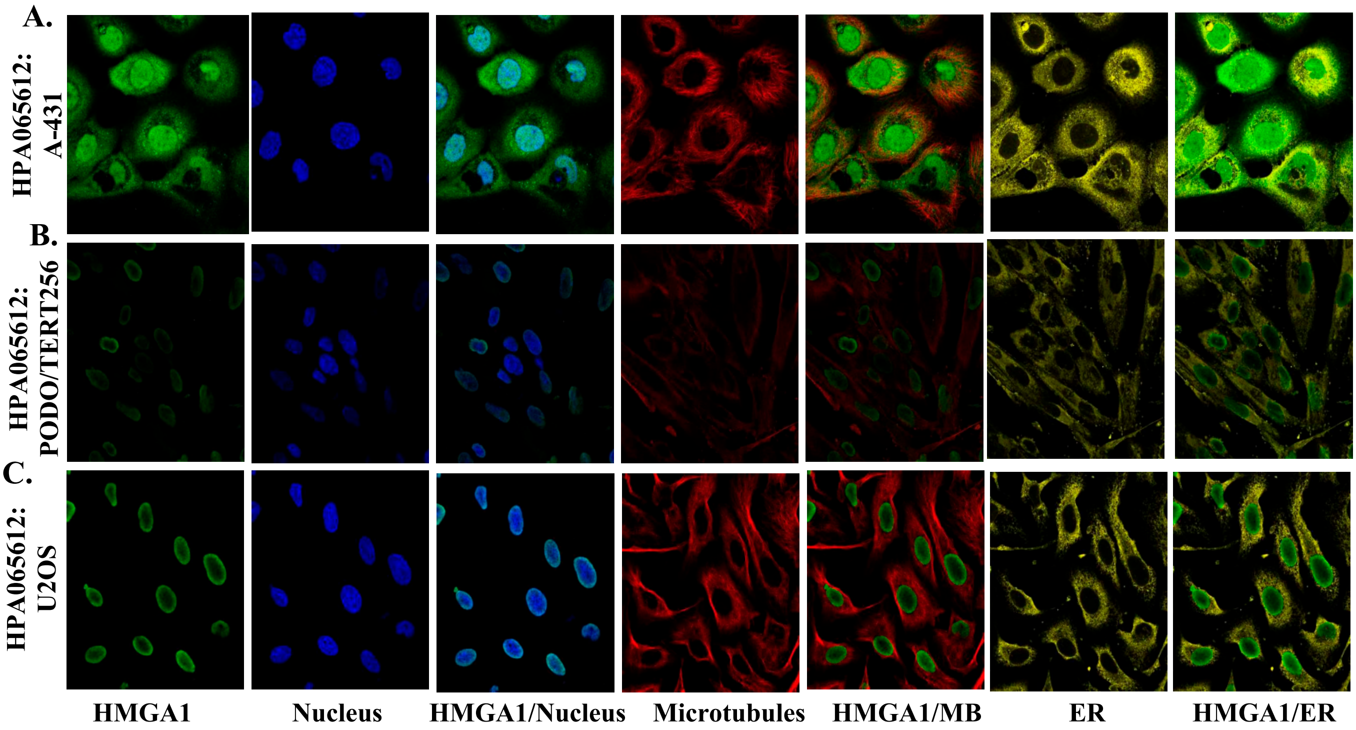

Supplementary Figure S2. Protein expression and subcellular localization of HMGA1. **(A)** Immunofluorescence images from the Human Protein Atlas (HPA) showing HMGA1 localization in A-431, PODO/TERT256, and U2OS cell lines, with predominant localization in the nucleoplasm and nuclear membrane, and additional presence in the cytosol.

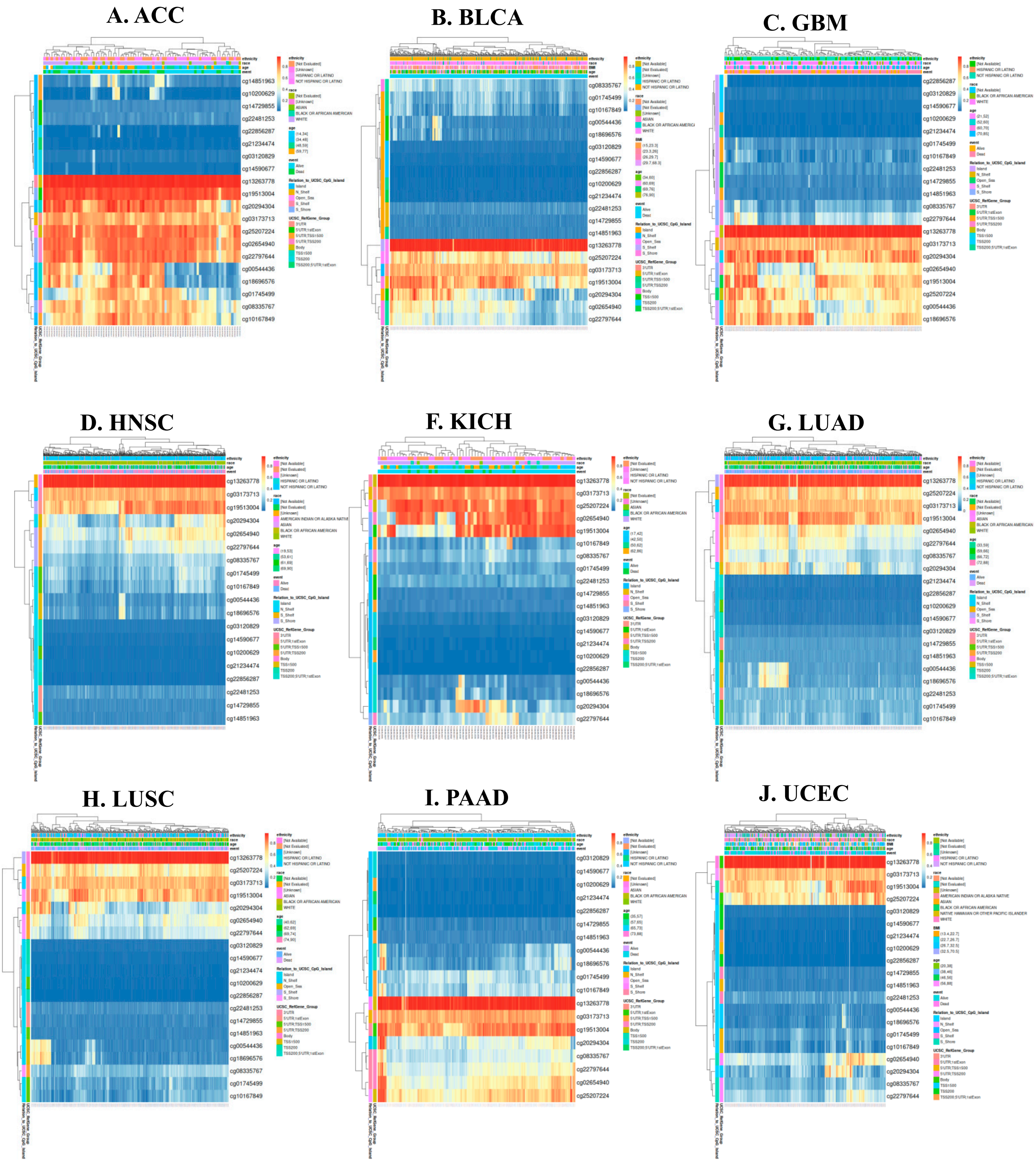

Supplementary Figure S3. DNA methylation analysis of HMGA1 promoter sites. **(A-J)** Distribution of DNA methylation at the HMGA1 locus in the representative cancers.

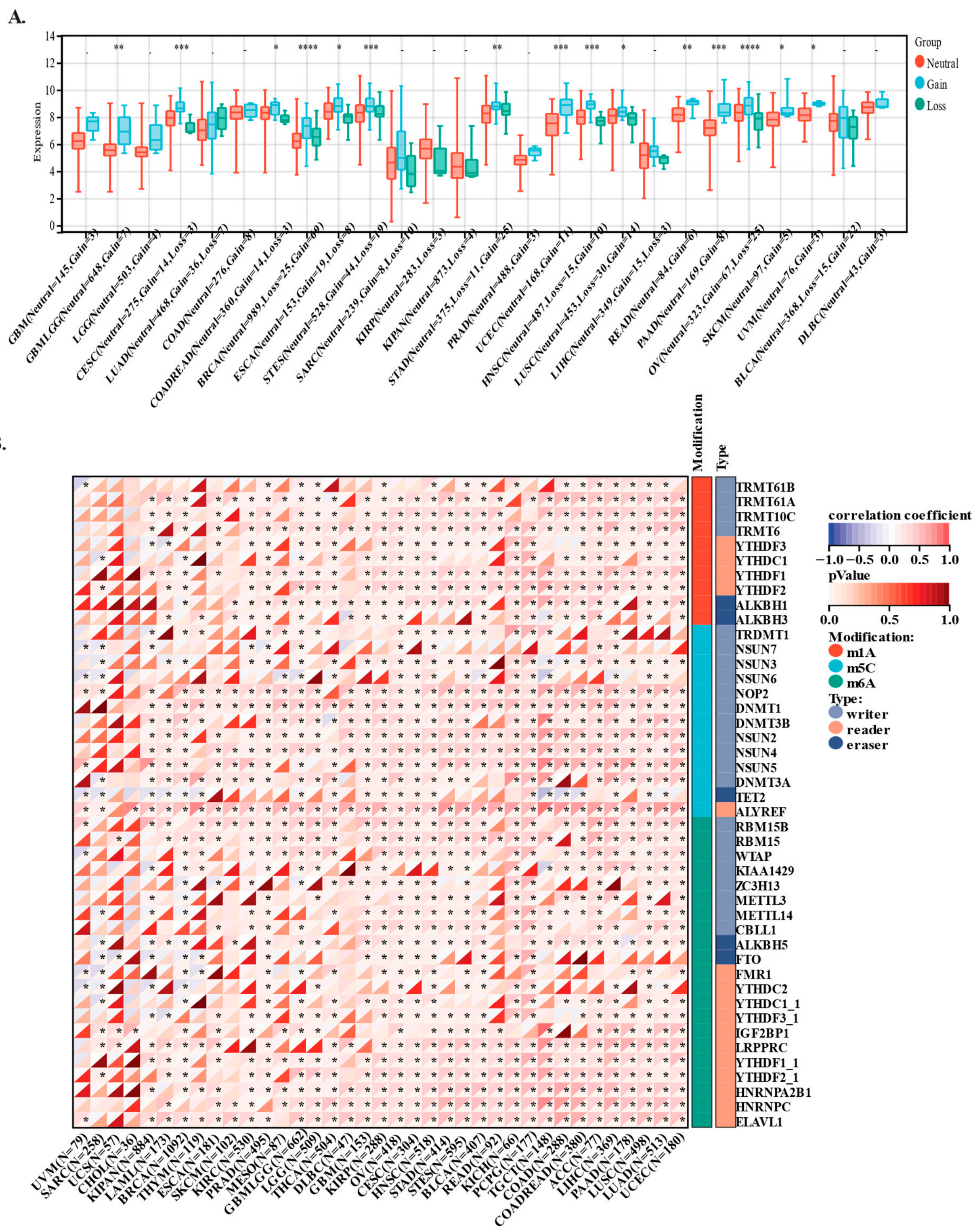

Supplementary Figure S4. Genetic Variations and Epitranscriptomic Correlations of HMGA1 **(A)** Boxplots show HMGA1 expression in samples classified as Neutral, Gain or loss across TCGA cancers. Statistical differences were determined by Wilcoxon or Kruskal- Wallis tests. ***p < 0.001; **p < 0.01; *p < 0.05; ns, not significant. **(B)** Heatmap showing Pearson correlation coefficients between HMGA1 and RNA modification-related genes, including m6A, m5C, and m1A regulators (classified as writers, readers, or erasers). Red indicates positive and blue indicates negative correlation.

A.

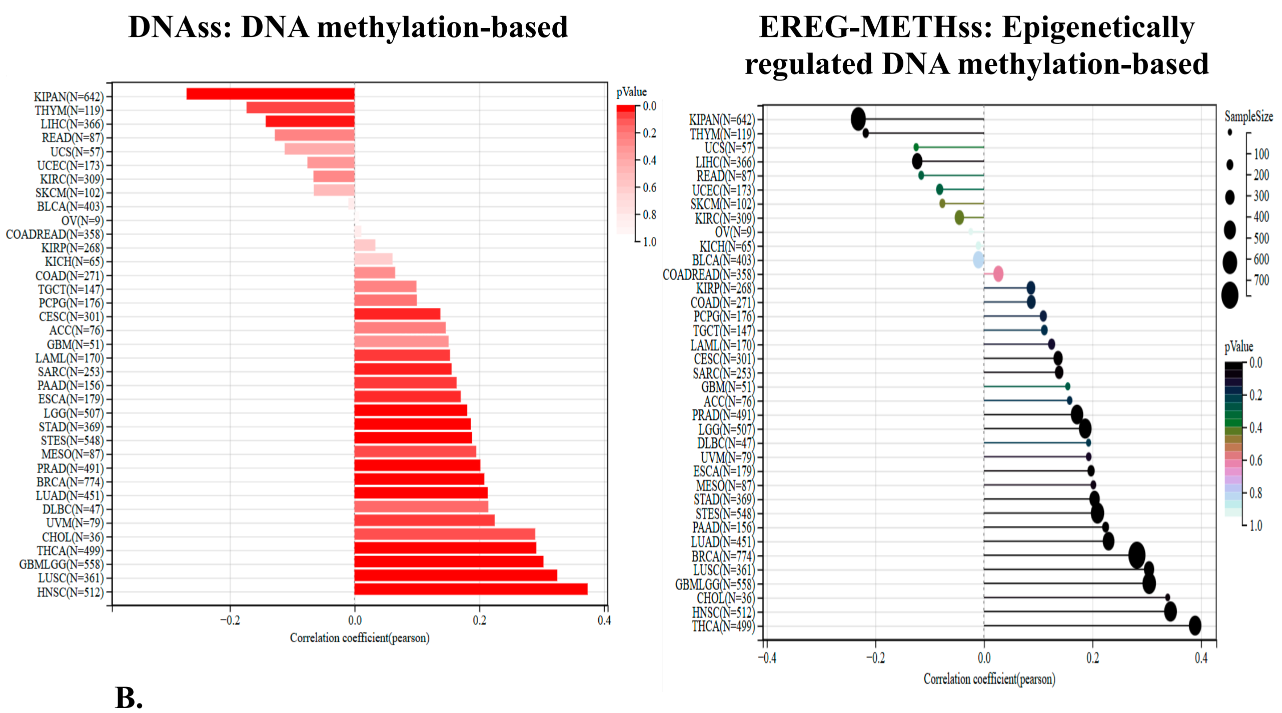

B.

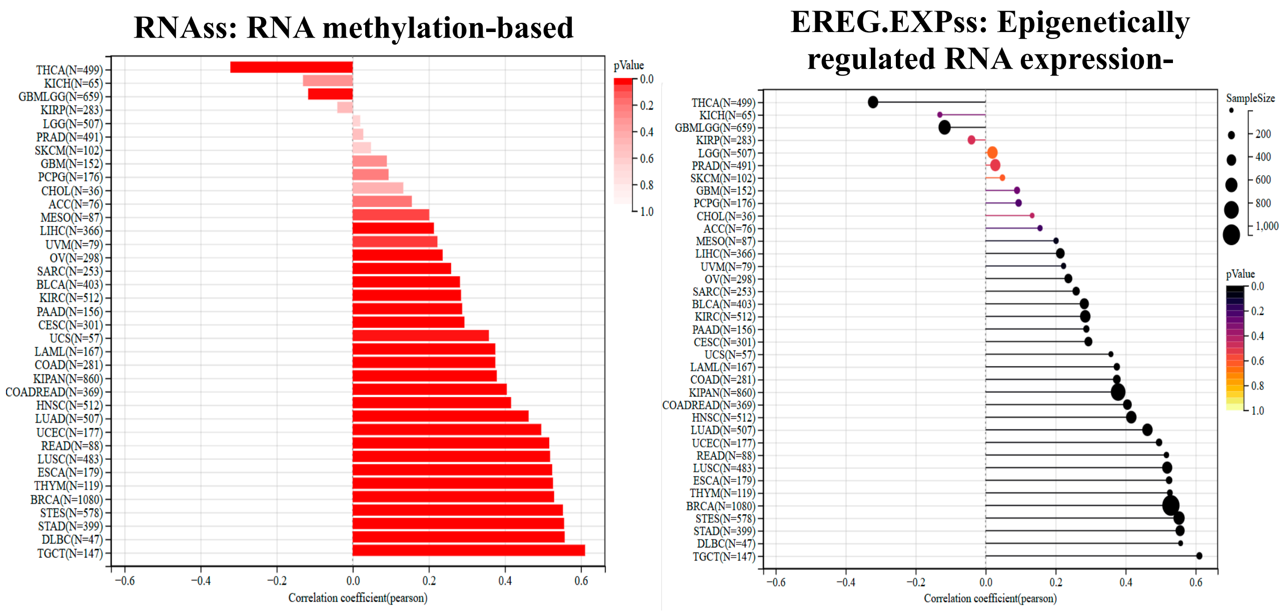

Supplementary Figure S5. (A-B) Correlations of HMGA1 expression with stemness indices (DNAss, RNAss, EREG-METHss, EREG-EXPSs) in representative cancers, showing strong positive associations.

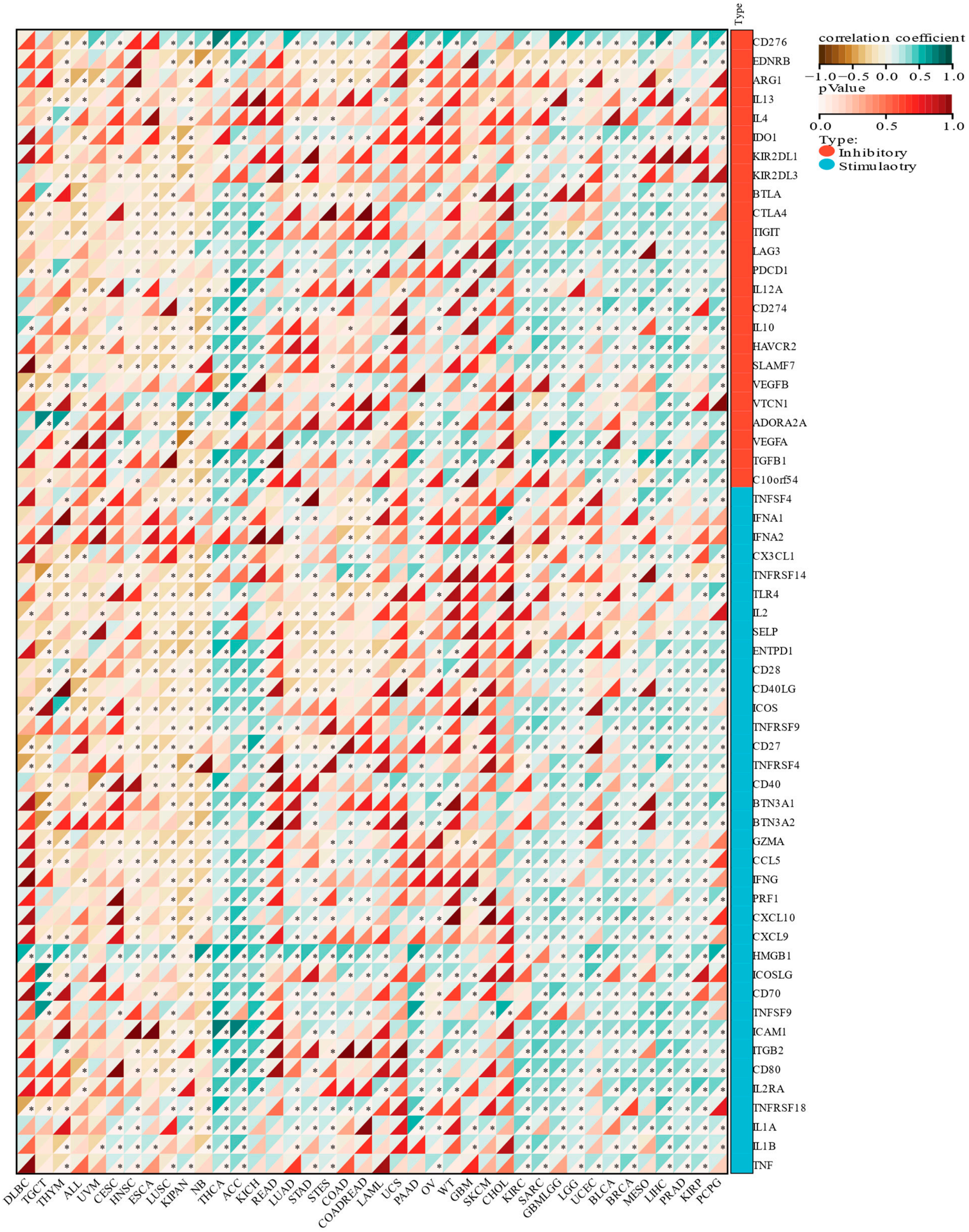

Supplementary Figure S6. HMGA1 correlation with immune inhibitory checkpoints and Treg-related markers in cancers.

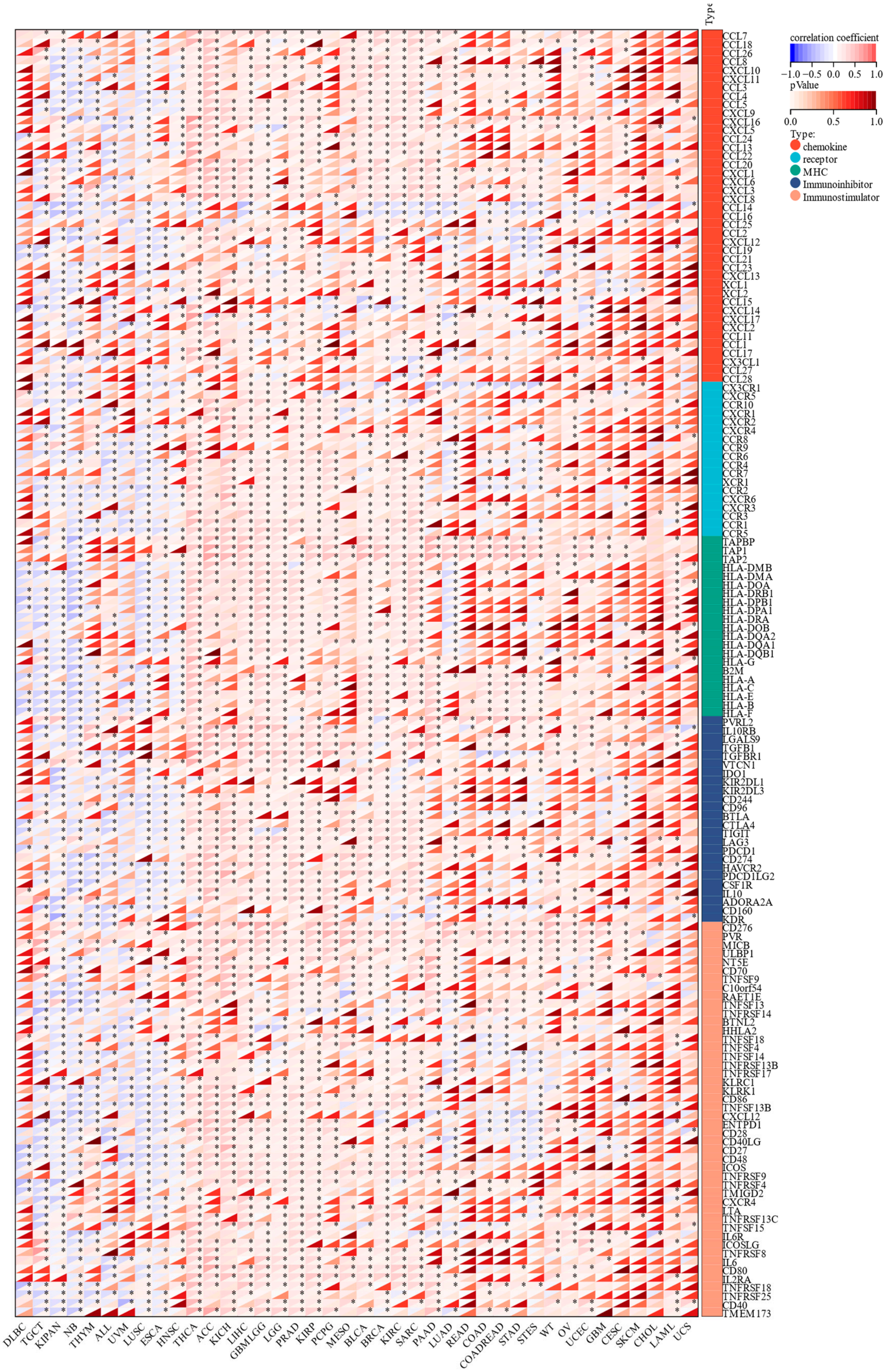

Supplementary Figure S7. Correlation of immune markers and chemokines with HMGA1 in cancers.

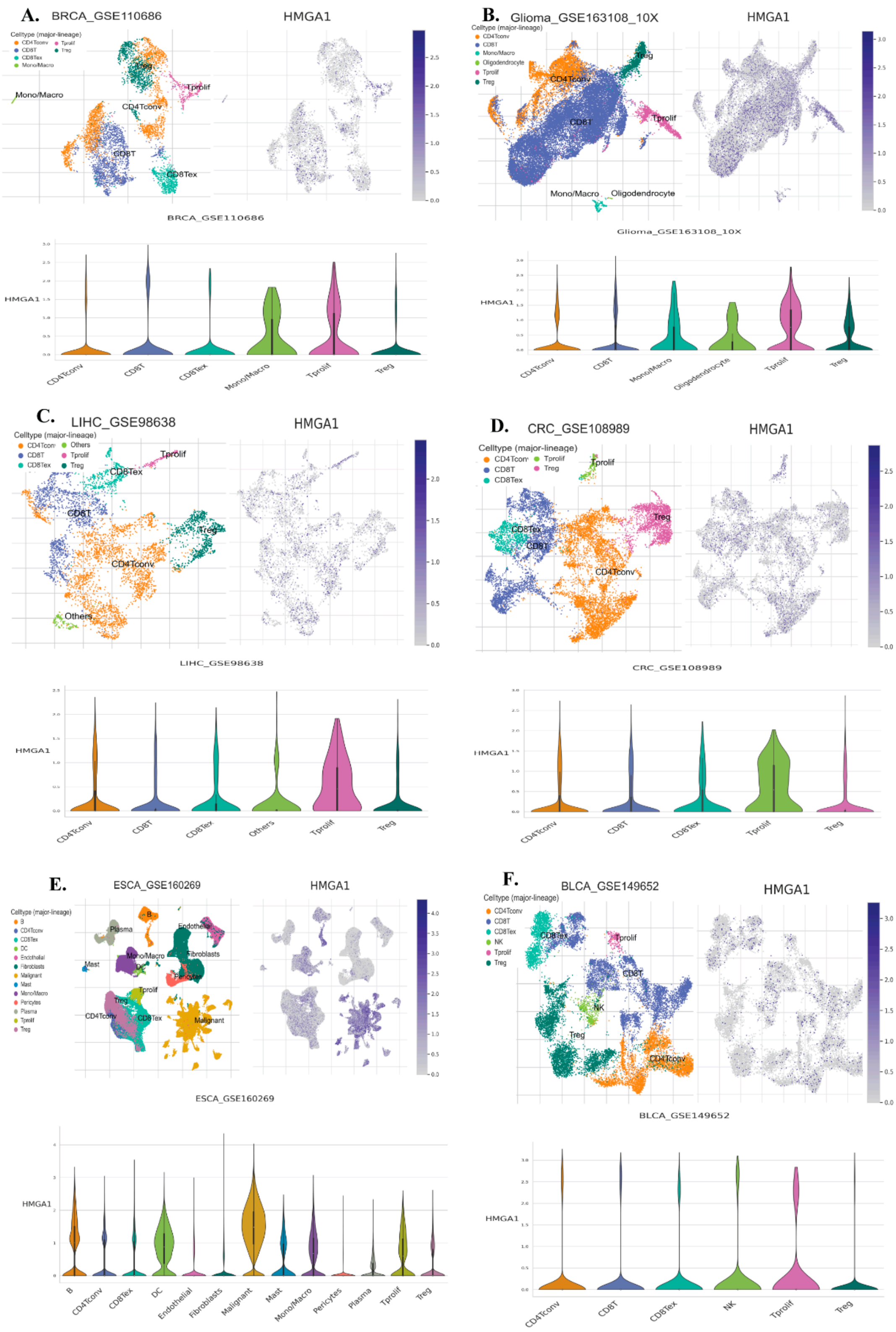

Supplementary Figure S8. Single-Cell expression landscape of HMGA1 across six cancer types **(A-F)** Single-cell RNA-sequencing data from TISCH demonstrate HMGA1 expression across major cellular compartments in Breast cancer **(A)**, Glioma **(B)**, Liver Hepatocarcinoma cancer **(C),** Colorectal cancer **(D),** Esophageal cancer **(E)**, and Bladder cancer **(F)**.

Supplementary Table S1: Cancers types abbreviations

Cancer Types	Abbrevations
TCGA-ACC	Adrenocortical carcinoma
TCGA-BLCA	Bladder Urothelial Carcinoma
TCGA-BRCA	Breast invasive carcinoma
TCGA-CESC	Cervical squamous cell carcinoma and endocervical adenocarcinoma
TCGA-CHOL	Cholangiocarcinoma
TCGA-COAD	Colon adenocarcinoma
TCGA-COADREAD	Colon adenocarcinoma/Rectum adenocarcinoma Esophageal carcinoma
TCGA-DLBC	Lymphoid Neoplasm Diffuse Large B-cell Lymphoma
TCGA-ESCA	Esophageal carcinoma
TCGA-FPPP	FFPE Pilot Phase II
TCGA-GBM	Glioblastoma multiforme
TCGA-GBMLGG	Glioma
TCGA-HNSC	Head and Neck squamous cell carcinoma
TCGA-KICH	Kidney Chromophobe
TCGA-KIPAN	Pan-kidney cohort (KICH+KIRC+KIRP)
TCGA-KIRC	Kidney renal clear cell carcinoma
TCGA-KIRP	Kidney renal papillary cell carcinoma
TCGA-LAML	Acute Myeloid Leukemia
TCGA-LGG	Brain Lower Grade Glioma
TCGA-LIHC	Liver hepatocellular carcinoma
TCGA-LUAD	Lung adenocarcinoma
TCGA-LUSC	Lung squamous cell carcinoma
TCGA-MESO	Mesothelioma
TCGA-OV	Ovarian serous cystadenocarcinoma
TCGA-PAAD	Pancreatic adenocarcinoma
TCGA-PCPG	Pheochromocytoma and Paraganglioma
TCGA-PRAD	Prostate adenocarcinoma
TCGA-READ	Rectum adenocarcinoma
TCGA-SARC	Sarcoma
TCGA-STAD	Stomach adenocarcinoma
TCGA-SKCM	Skin Cutaneous Melanoma

TCGA-STES	Stomach and Esophageal carcinoma
TCGA-TGCT	Testicular Germ Cell Tumors
TCGA-THCA	Thyroid carcinoma
TCGA-THYM	Thymoma
TCGA-UCEC	Uterine Corpus Endometrial Carcinoma
TCGA-UCS	Uterine Carcinosarcoma
TCGA-UVM	Uveal Melanoma
TARGET-OS	Osteosarcoma
TARGET-ALL	Acute Lymphoblastic Leukemia
TARGET-NB	Neuroblastoma
TARGET-WT	High-Risk Wilms Tumor

Supplementary Table S3. sc-RNA Sequencing Datasets

Species	Tumor	Tissue	Sequence	Cells	Sample	PubMed ID
Human	BRCA_GSE110686	Breast	10X Genomics	6035	2	29942092 Savas P,et al. (2018)
Human	GBM_GSE163108	Glioma	10X Genomics	25013	5	33592174 Mathewson et al. (2021)
Human	LIHC_GSE98638	Liver	Smart-Seq2	5059	6	28622514 Zheng C,et al. (2017)
Human	CRC_GSE108989	Colon	Smart-Seq2	11125	12	30479382 Zhang L,et al. (2018)
Human	ESCA_GSE160269	Esophagus	10X Genomics	208658	64	34489433 Zhang X,et al. (2021)
Human	BLCA_GSE149652	Bladder	10X Genomics	15538	7	32497499 David,et al. (2020)
